# Menoprogen, a TCM Herbal Formula for Menopause, Increases Endogenous E_2_ in an Aged Rat Model of Menopause by Reducing Ovarian Granulosa Cell Apoptosis

**DOI:** 10.1155/2016/2574637

**Published:** 2016-02-14

**Authors:** Yu Li, Hong Ma, Ye Lu, B. J. Tan, L. Xu, Temitope O. Lawal, Gail B. Mahady, Daniel Liu

**Affiliations:** ^1^Traditional Chinese Medicine Department, Nanjing University of Chinese Medicine, Nanjing 210046, China; ^2^Institute of Botany, Chinese Academy of Science, Nanjing, Jiangsu 210014, China; ^3^Hainan Medical College, Haikou, Hainan 571101, China; ^4^Department of Pharmacy Practice, College of Pharmacy, University of Illinois at Chicago, Chicago, IL 60612, USA; ^5^Beijing Clinical Services Center, No. 103 Chaoyang North Road, Beijing 100123, China

## Abstract

The effect of Menoprogen (MPG) on ovarian granulosa cell (GC) apoptosis was investigated in vitro and in vivo in an aged rat model of menopause. Intragastric administration of Menoprogen or estradiol valerate to 14-month-old senile female rats for eight weeks increased plasma E_2_ levels, as well as the weight of both ovarian and uterine tissues. Flow cytometric (FCM) analysis of isolated GCs from MPG-treated aged rats showed reductions in the G_0_/G_1_ ratio and apoptotic peaks. Isolated GCs also exhibited an increase in cell size and the number of cytoplastic organelles and intracellular gap junctions, the reappearance of secretory granules, and a lack of apoptotic bodies as determined by TEM. Results from a TdT-mediated dUTP nick end-labeling (TUNEL) assay revealed a reduction in TUNEL-positive GCs after MPG treatment. Immunohistochemical analysis showed a downregulation of proapoptotic Bax proteins and an upregulation of antiapoptotic Bcl-2 proteins. The addition of MPG-medicated serum to the media of cultured GCs also reduced cadmium chloride-induced apoptosis and downregulated caspase-3 protein expression. This work demonstrates that Menoprogen inhibits GC apoptosis in aged female rats and thereby increases E_2_ production. This represents a novel mechanism of action for this herbal medicine in the treatment of menopausal symptoms.

## 1. Introduction

Menopause is defined as the permanent cessation of menstruation (≥12 months), resulting from the loss of ovarian follicular activity [1, 2]. It is preceded by the menopausal transition (perimenopause), which is associated with erratic hormone levels and diverse clinical symptoms [3]. The symptoms associated with the menopause and its transition are due to alterations within the ovary, of which the most prominent change is a dramatic decline in follicle numbers due to ageing [4, 5]. At the cellular level, ovarian follicle ageing and reproductive senescence are characterized by a decline in specific functions of oocytes and granulosa cells (GCs) due to generalized cellular dysfunction, including a reduction in mitochondrial activity and energy failure, as well as increases in the expression of senescence genes and proteins [4, 5]. The degree of cellular decline that occurs with ageing is sufficient to increase the susceptibility of the ovarian follicles, ovulated oocytes, and granulosa cells to apoptosis [4, 5], and it is GC apoptosis that triggers follicular atresia [6]. Thus, apoptosis is a primary driving force behind follicle loss during ageing, causing the age-related decline in the function of oocytes and granulosa cells [4–6]. Since GCs are the primary source of estradiol and progesterone synthesis in the ovary, a reduction in their number and function leads to reduced levels of endogenous hormones and, hence, menopausal symptoms [5, 6].

One of the most problematic issues associated with declining ovarian function is that of lowered estrogen (E_2_) levels [1]. The decline in E_2_ causes a neuroendocrine imbalance and menopausal symptoms, such as hot flashes, headaches, nocturnal sweating, vaginal atrophy, and anxiety. Chronically lower E_2_ levels also lead to significantly higher incidences of cardiocerebrovascular diseases, osteoporosis, and senile dementia in postmenopausal women [1]. In the past, hormone therapy (HT) has been the “gold standard” for the treatment of menopause. However, the use of HT worldwide has declined due to an increased risk of adverse events, including a transitory increased risk of heart disease, and increased risk of endometrial or breast cancer [7]. On the basis of the findings and risk analyses of the Women's Health Initiative, the US Preventive Services Task Force has recommended* against* the routine use of HT for the prevention of chronic conditions in postmenopausal women [7]. As a consequence, many women have been looking for complementary and alternative medicines, including Traditional Chinese Medicines (TCM), to manage their menopausal symptoms.

One TCM formula, Menoprogen, has been marketed as an herbal supplement in China and Europe for over 15 years and has been available in the USA for about ten years for the management of menopausal symptoms [8, 9]. Menoprogen is an ancient TCM formula containing semipurified extracts of five well-known “herbal food” plants, which have been used as dietetic and medical herbs in China for thousands of years [8–11]. The herbal formula is comprised of extracts of the herbs Fructus Lycii (*Lycium barbarum* L., fruits, Chinese goji berries, Solanaceae); Semen Cuscutae (*Cuscuta chinensis* Lam., ripe seeds, Convolvulaceae), Radix Rehmanniae (*Rehmannia glutinosa* Libosch., roots, Scrophulariaceae), Fructus Mori (*Morus alba* L., white mulberry fruits, Moraceae), and Flos Carthami (*Carthamus tinctorius* L., safflower, Asteraceae) [8–11]. In two pilot clinical trials, Menoprogen (MPG) was shown to be effective for the management of menopausal symptoms and reduced the Kupperman index in treated women [8, 9]. In animal studies, oral administration of MPG to aged female rats restored ovarian function [12, 13]. Furthermore, oral administration of MPG to female rats also increased blood circulation to the ovary and uterine tissues as well as improved the quality of ovarian follicles [14]; however the mechanism of action for MPG has not been fully elucidated. In this work, we investigate the effects of Menoprogen in aged female rats and provide evidence that this herbal formula reduces GC apoptosis through a mechanism involving the Bcl-2/Bax family of proteins and caspase-3. The reduction in GC apoptosis increases the production of endogenous E_2_, thereby demonstrating a novel mechanism of action for this herbal formula.

## 2. Materials and Methods

### 2.1. Drugs and Reagents

The MPG herbal formula was provided by Nanjing Mayflower Pharmaceutical Technology Corporation Ltd. (Nanjing, China) and contains five Chinese herbal extracts as described above [8, 9]. An optimized high-performance liquid chromatography-photodiode array detection (HPLC-PDA) fingerprinting method for MPG was developed and has shown that some of the primary chemical constituents of MPG are flavonoids [9]. Progynova (estradiol valerate tablets) were used as a positive control and were obtained from Schering SA Company, France. Pregnant mare serum gonadotropin (PMSG) was purchased from Chuangrui Biological Reagent Company (Nanjing, China); cadmium chloride from Tingxin Chemical Pharmaceutical Company (Shanghai, China); glutaraldehyde phosphate buffered solution (PBS) from Boster Company (Wuhan, China); lead citrate trihydrate and uranyl acetate dyeing solution from China National Pharmaceutical Group Corp. (Shanghai, China); PI dyeing solution from Sigma (St. Louis, MO, USA); and fetal bovine serum from Gibco (USA).

The radioimmunoassay kit for the serum estrogen assay was obtained from Diagnostic Systems Laboratories Inc. (Webster, TX, USA); TdT-mediated dUTP nick end-labeling (TUNEL) kit was obtained from Roche Diagnostic GmbH (Sandhofer, Mannheim, Germany); immunochemical assay kit for Bax and Bcl-2 from Santa Cruz Biotechnology (Santa Cruz, CA, USA); and enzyme-linked immunosorbent assay (ELISA) kit for caspase-3 from Biyuntian Biological Reagent Company (Nantong, China).

### 2.2. In Vivo Rat Model of Naturally Aged Female Rats (NAM)

A total of 60 Sprague-Dawley female rats (SPF grade) and feed were purchased from B&K Universal Group Limited (Shanghai, China). All rats were fed an aseptic diet ad libitum under controlled conditions 24°C, 50–55% humidity, and a 12-hour light/dark cycle. The animal care protocols were approved in accordance with the UK Animal Scientific Procedures Act of 1986 and associated guidelines. All protocols were approved by the ACC prior to initiation of the research study. For two continuous estrous cycles, fifty 14-month SD female rats were monitored by a smear test of exfoliated vaginal epithelial cells, and the blood was drawn intraorbitally for the determination of E_2_ in the serum. Rats with continuously irregular estrous cycles and a significant decrease in E_2_ levels were regarded as naturally aged model (NAM). The NAM rats were randomly divided into 5 groups: NAM group (*n* = 10), MPG low-dose treatment group (162 mg/kg body weight, *n* = 10), MPG middle-dose treatment group (324 mg/kg, *n* = 10), MPG high-dose treatment group (648 mg/kg, *n* = 10), and positive estradiol valerate control (Progynova, *n* = 10) group. In addition, 4-month-old female rats (*n* = 10) were selected as a normal control group. Rats in the MPG treatment groups were treated with an aqueous extract of Menoprogen at equivalent clinical dosages (648, 324, and 162 mg/kg p.o., resp.), while rats in the positive control group were treated orally with estradiol valerate (Progynova, dose 0.18 mg/kg). All rats underwent a smear test of exfoliated vaginal epithelial cells after 8-week treatment. Following treatment, after being weighed, but before estrous cycle and blood sampling, the rats were sacrificed after anesthetization. The bilateral ovaries and uterus were removed, and the wet weight was recorded. The comparison of organ weight was performed using an organ coefficient, which was calculated according to following formula: organ coefficient = organ wet weight/body weight × 100%. One of the bilateral ovaries was used for the analyses by transmission electron microscope (TEM) and flow cytometry (FCM), and another was fixed in 10% neutral formalin for 24 h, gradually dehydrated by alcohol, transparentized with xylene, and then embedded in paraffin for the TUNEL assay and the Bcl-2 and Bax immunohistochemical assay.

### 2.3. Analysis of Serum E_2_

Serum samples were processed by a centrifugation at 3000 rpm for 15 min followed by the measurement of E_2_ by radioimmunoassay (Diagnostic Systems Laboratories Inc., Webster, TX, USA) using the protocol described by the manufacturer.

### 2.4. Transmission Electron Microscopy (TEM)

One to three turbid follicles were removed from a unilateral ovary (soaked in saline) using an ophthalmic scissor, and then the follicles were pricked using a 28-gauge needle to drain follicle fluids. The follicles were first fixed in 5% glutaraldehyde and then in 1% PBS (pH = 7.4, 4°C) and were processed under gradient dehydration with acetone and ethanol and embedded using Epon 812 and epoxy resins. The embedded blocks were trimmed and located by a repairing instrument and then sliced in 50 nm thickness by ultramicrotome. The slices were polymerized and stained with lead citrate and uranyl acetate. The observation was conducted and photographed using a TECNAI 12 TEM (Philips, Netherlands).

### 2.5. Assessment of Apoptosis by Flow Cytometry (FCM)

New turbid follicles were excised from the remaining unilateral ovary and were washed twice with sterile normal saline (NS) at 37°C. Granulosa cells were isolated from the medium (2–5 mm diameter in size) antral follicles by fine-needle aspiration for FCM analysis as described. The saline solution containing the GCs was centrifuged at 1000 rpm for 10 min. The pellet was rinsed twice with NS and fixed in 75% ice-cold ethyl alcohol. After the fixed GCs were centrifuged at 1000 rpm for 10 min, the supernatants were removed. The sediment was rinsed with NS and then resuspended in 0.5 mL NS and filtered through 400-mesh nylon membrane. The resulting solution was stained with propidium iodide in dark at 4°C for 30 min and then assayed for DNA contents in cells at each stage by FCM analysis. The data was analyzed by Cell Quest and ModiFit 2.0 (USA) and described in a DNA histogram.

### 2.6. Assessment of Apoptosis Using the TUNEL Assay

The TdT-mediated dUTP nick end-labeling (TUNEL) assay was performed using the protocol of the manufacturer (described above). Briefly, paraffin slices in 4 *μ*m thickness were dried for 2 h in oven at 80°C. After conventional dewaxing, the samples were set with citrate buffer (1 : 2000) in microwave oven for 8 min and washed with PBS (0.01 M, pH 7.4) three times for 2 min each time. All slices were dried, digested with protease K 50 *μ*L for 15 min, washed by PBS, and then dried. The slices were then placed in 0.1% triton at room temperature for 8 min, washed, and dried again; an enzyme solution and a nucleotide reaction solution were added. Having been subsequently incubated at 37°C for one hour, the slices were washed, dried, and then incubated with enzyme-labeled anti-fluorescence antibody at 37°C for an additional 30 min. After DAB color development for 10 min, the reacted slices were then washed with distilled water, transparentized with xylene, sealed by resin adhesive, and finally measured under light microscope.

### 2.7. Immunohistochemistry

Paraffin slices were dewaxed, trimmed, washed with PBS, and sealed by 10% normal goat serum. The slices were rinsed in buffer before the primary bcl-2 (Cat. number M0887, clone 124, Dako Corporation, CA; 1 : 100 dilution) or bax (Cat. number 18-0218, clone 2D2, Zymed Laboratories Inc., San Francisco, CA; 1 : 200 dilution) antibodies were overlaid at 37°C as previously described [15]. The reacted slices were washed and incubated with the immunohistochemical kit MaxVision*™* for additional 15 min at room temperature, according to the instructions of the manufacturer. Counterstaining was then performed using DAB (3,3′-diaminobenzidine) which is the most commonly used amine substrate for histochemical detection of cell surface and intracellular antigens on frozen or embedded tissue sections, and routinely used to stain rodent tissue sections. DAB solution was prepared by adding 5 mL of double distilled water (ddH_2_O), 3 drops of Tris buffer (ca. 150 *μ*L), 2 drops of DAB solution (ca. 100 *μ*L), and 2 drops of peroxide solution and kept in the dark. The DAB solution was applied to the section and incubated for 3–5 min under a cover of aluminum foil. The development of the color was then observed under a microscope and immediately stopped by removing the DAB and rinsing the section with ddH_2_O for at least 1 min. The sections were counterstained with hematoxylin and mounted with xylene. After DAB color development and hematoxylin counterstaining, the slices became blue following washing with distilled water. After the gradient dehydration with alcohol, the slices were transparentized by xylene and then sealed by resin adhesive.

### 2.8. Preparation of Serum for In Vitro Model of GCs

Menoprogen was administered daily to Sprague-Dawley rats (200 ± 20 g; *n* = 8) by gavage at a clinical dosage of 648 mg/kg body weight (representing 10 times the dose for an adult of 60 kg, which equals the everyday therapeutic dosage of an adult × animal-human dosage exchange ratio × 10) [16]. The control group was treated with equal volume of saline each time for three days. After three days of treatment, abdominal aortic blood was collected before the rats had fasted for 12 hours. Then blood samples were centrifuged for 15 minutes at 3000 rpm and inactivated in 56°C for 30 minutes. Nonmedicated serum and medicated serums were sterilized by microporous membrane and stored at −80°C until used.

### 2.9. In Vitro Induction of Apoptosis in Granulosa Cells (GCs) by Cadmium Chloride

Cadmium chloride (CdCl) is a heavy metal that can induce cellular apoptosis via the caspase and Bcl-2 pathways [17, 18]. We have used this heavy metal to induce apoptosis in GCs and then determine the effect of MPG on CdCl-induced apoptosis. Three SD female rats at age of 22–27 days were sacrificed 48 hours after injection with 50 IU pregnant mare's serum gonadotropin (PMSG). The bilateral ovaries split from capsules and peripheral adipose tissues were obtained and washed twice with normal saline. In the DMEM-F12 culture media containing 10% calf serum, the follicular membrane was broken by ophthalmological scissor to release the GCs [19]. The cell number was counted using a hemocytometer under phase-contrast microscopy and viability was assessed using trypan blue dye exclusion. The solution with GCs was stirred and filtered through 400-mesh nylon filter membrane. After centrifugation at 1000 rpm for 5 min, the GCs solutions were rinsed twice and resuspended in the culture media. The GCs suspension was adjusted to 2 × 10^6^ cells/mL in the culture media and incubated under 5% CO_2_, at 37°C for 2 hours. Cells in the low-dose group, middle-dose group, high-dose group, serum control group, and model group were cultured with various concentrations of MPG-containing serum (25%, 50%, and 100%), normal mouse serum, and 40 *μ*mol/L cadmium chloride (CdCl) for 24 hours [19], respectively.

### 2.10. MTT Assay of Cultured GCs

An MTT (3-(4,5-dimethylthiazol-2-yl)-2,5-diphenyltetrazolium bromide) cell viability assay kit was used to measure GC proliferation (Sigma-Aldrich, USA). After incubation under 5% CO_2_ at 37°C for 24 h, GCs (10^5^ cells per well) were subsequently inoculated onto 96-well plates. After treatment for 24 h, the supernatant was discarded, 100 *μ*L DMSO was added, the 96-well plate was mixed on microvibrator for additional 10 min, and the optical density of each well was measured at *λ*570 nm using an enzyme-immunoassay plate reader (Biorad, USA). The experiment was performed in triplicate and an average value was used to determine the final result after the measurement was repeated three times.

### 2.11. TEM of Cultured GCs

For TEM observation, cultured GCs were collected and fixed with 2% (v/v) glutaraldehyde in PBS for 2 h and then with 2% (v/v) osmium tetroxide in phosphate buffer for additional 2 h. Thin sections were prepared and stained as described in a previous study [20]. The sections were observed using a JEM1230 TEM (JEOL USA, Peabody, MA).

### 2.12. Assessment of Apoptosis in Cultured GCs by Flow Cytometry

Cultured GCs were fixed in 75% ice ethyl alcohol and centrifuged for 10 min at 2000 rpm. After the supernatant was discarded, the pellets were rinsed twice with NS and then resuspended in 0.5 mL NS. The suspension was filtered through 400-mesh nylon filter membrane and stained with PI dyeing solution in dark for 30 min. FCM was used to analyze the DNA content of cells at different stages, and data was analyzed as described above.

### 2.13. Caspase-3 Analysis in Cultured GCs

The Capase-3 kit was used according to the manufacturer's instructions (Biyuntian, Nantong, China). Briefly, a *p*-nitroanilide (pNA) standard curve was generated; then increasing concentrations of the drug-containing serum and CdCl were added to the cultured GCs. After 2 hours of treatment, the cells were digested and centrifuged (1000 rpm, 10 min) after 24 h; supernatant was removed while the cell pellet was washed twice with PBS and centrifuged again. The pellets were suspended in the lysate solution at a ratio of 100 *μ*L lysate to 2 million cells. The GCs were then lysed in an ice bath for 15 min. The supernatants, which were obtained by centrifugation at 14,000 rpm at 4°C for 15 min, were placed on ice bath for further analysis. The caspase-3 concentration was measured using the protocol described by the manufacturer. After color development, the OD value was measured at 405 nm using an enzyme immunoassay plate reader. The concentrations were derived from the predefined standard curve. The immunochemical assay kits for Bax and Bcl-2 (described above) were used according to the instructions of the manufacturer.

### 2.14. Statistical Analysis

All of the data were expressed as mean ($\overset{-}{x}$) and standard deviation (sd) and were analyzed using one-way ANOVA followed by Dunnett's test for the post hoc analysis using the statistical software SPSS 12.0 (CA, USA).

## 3. Results

### 3.1. Effects of Menoprogen on Ovarian and Uterine Weights of Aged Female Rats (NAM)

Compared with normal four-month-old control female rats, the 14-month-old NAM rats exhibited ovarian and uterine atrophy and exhibited significant decreases in the ovarian and uterine coefficients (described in Section 2), respectively (Table 1). Both the ovarian and uterine coefficients in Menoprogen-treated aged rats significantly increased, while only the uterine coefficient increased in the Progynova-fed rats.

### 3.2. Menoprogen Enhances Serum E_2_ Concentrations

As shown in Table 2, the serum E_2_ concentrations of the 14-month-old NAM rats were significantly lower than that of 4-month-old control rats, indicating a decline of reproductive endocrine functions that is commonly observed during the natural ageing process. The serum E_2_ levels showed significant increases after oral administration of Progynova or Menoprogen to the NAM rats (Table 2).

### 3.3. Effects of Menoprogen on Ovarian GC Ultrastructure from NAM Rats

**Figure 1:**
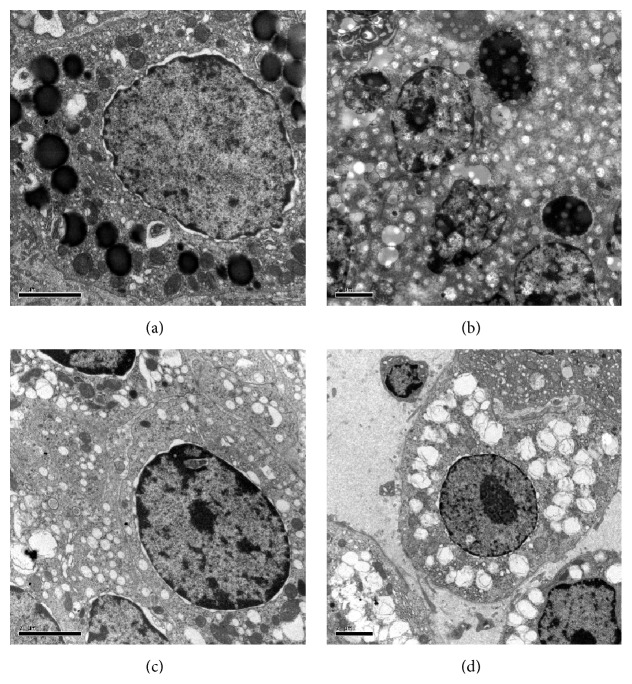
The effects of Menoprogen and Progynova on ovarian GC ultrastructure. (a) Euchromatins were observed in the normal control group, and all cell organs were morphologically normal. (b) In the NAM group, GC nuclei exhibited smaller cell bodies, concentrated chromatins, and apoptotic cells. (c) GCs in the Menoprogen group also had increased euchromatins as compared with the NAM control rats, cellular organs were increased, and there were no apoptotic bodies observed. (d) In the Progynova group the GCs displayed primarily euchromatin and a few apoptotic bodies were observed.

A series of electron micrographs showing the ovarian GC ultrastructure pattern as observed under TEM are presented in Figure 1. As seen in Figure 1(a), the nucleus of GCs isolated from normal control rats is round or oval and contained more euchromatin, indicating higher gene transcription. These normal GCs also exhibited an increase in vesicle-like smooth endoplasmic reticulum (SER), scattering distribution of mitochondria with normal tubular cristae, and more Golgi complexes and round lipid droplets.

In contrast to the normal controls, the nucleus of GCs from NAM rats exhibited more heterochromatin with marginal concentration, agglomeration and pyknosis in crescent or fragment, and higher nucleocytoplasmic ratio (Figure 1(b)). The abnormally concentrated cytoplasms contained fewer SERs and mitochondrion, smaller mitochondrion, disorganized structures of Golgi complexes, higher number of apoptotic cells and bodies, and few secretory granules. Furthermore, there was a broadening of sinusoidal gaps and certain myelin-like deformation between, and within GCs were found (Figure 1(b)).

The ovarian GC nuclei from the Menoprogen-treated NAM rats exhibited increased quantities of euchromatin and cellular organelles, including more SERs with round dilation, redundant mitochondria, and more normally structured Golgi complexes. Some secretory granules and sparse apoptotic cells were seen, but no apoptotic bodies were observed (Figure 1(c)).

The GC nucleus of Progynova-fed rats primarily displayed euchromatin, abundant cytoplasmic mitochondria, SERs, Golgi complexes and secretory granules, and a few of apoptotic cells and apoptotic bodies. Moreover, more cytoplasmic residual vacuoles and wider sinusoidal gap between GCs were observed (Figure 1(d)).

### 3.4. Effects of Menoprogen and Estradiol Valerate on the GC Cell Cycle

Isolated ovarian GCs from untreated and untreated NAM rats were subjected to FCM. The results demonstrated that when compared with the untreated NAM rats, both the normal control animals and MPG-treated NAM rats (treated with the middle or high dose of MPG or E_2_) had a lower percentage of GC cells in the G_0_/G_1_ stage (Table 3). In addition, more GCs were in the S stage in normal control and MPG-treated rats (low and middle dose). More GCs from the normal control rats were in the G_2_/M phase of the cell cycle (Table 3).

### 3.5. TUNEL and TEM Assessment of Follicles

**Figure 2:**
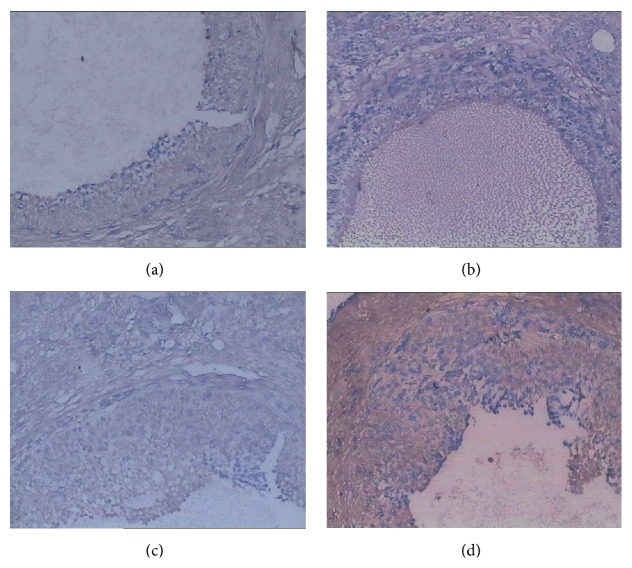
Effects of Menoprogen on GC apoptosis using the TdT-mediated dUTP nick end-labeling (TUNEL) assay. Apoptotic cells were detected as TUNEL-positive cells (brown staining). (a) Light microscopy showed many developing follicles at different stages and corpora lutea in normal control group. (b) In the NAM group there were more atretic follicles and apoptotic expression was prominent. (c, d) There were more developing follicles at different stages in the Menoprogen-treated (c) group and the Progynova group (d) (200x).

TUNEL and TEM assessment of follicles from normal control rats showed many developing follicles at different stages and corpora lutea, including large mature follicles full of follicular fluids (Figure 2(a)). Protuberant oocytes shaped cumulus oophorus in follicular cavity, and the follicles were hunching on the ovarian surface. The NAM rats had fewer developing follicles and corpora lutea, but more atretic follicles (Figure 2(b)). Apoptotic expression in atretic follicles and corpora lutea was much more prominent in the untreated NAM rats than those of the normal control rats. The MPG-treated rats showed an increased restoration of developing follicles at different stages and numbers of corpora lutea and less atretic follicles (Figure 2(c)). The Progynova-treated NAM rats exhibited higher quantities of both developing follicles and atretic follicles (Figure 2(d)).

### 3.6. Effects of Menoprogen on Expression of Bax/Bcl-2 Proteins

**Figure 3:**
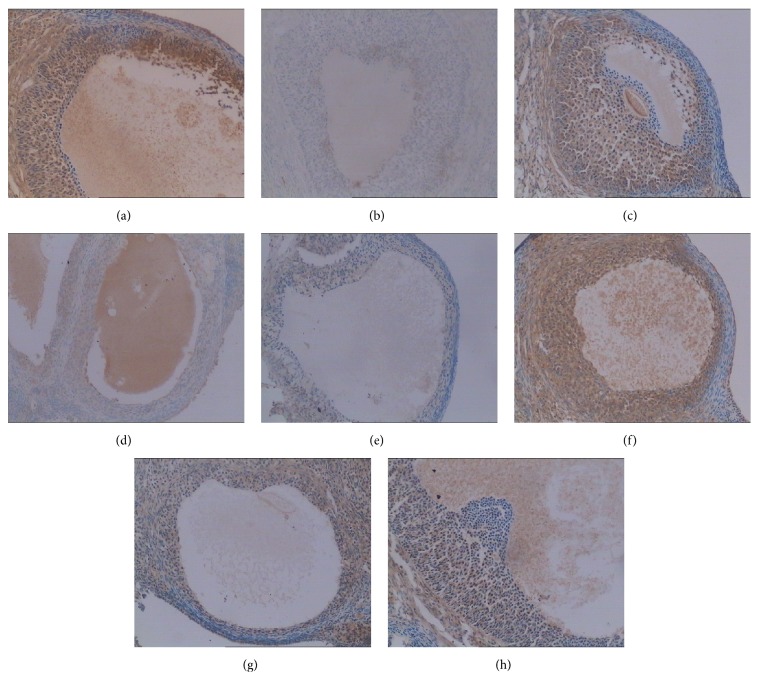
Effects of Menoprogen on expression of Bcl-2 and Bax proteins. (a) There was higher Bcl-2 protein expression in the normal control group (b) and lower Bcl-2 expression in the untreated NAM group. (c) The expression of Bcl-2 was increased in NAM rats after treatment with Menoprogen. (d) When compared with the Menoprogen treated rats, the expression of Bcl-2 was higher in the Progynova treated rats. (e) In addition, the normal control group showed a weak expression of Bax protein, (f) while the untreated NAM group showed strongly positive expression of Bax. (g, h) The expression of Bax protein decreased in both the Menoprogen treated (g) and the Progynova treated (h) groups (200x).

Bcl-2 is an antiapoptotic gene, and its protein is present as a buffy particle with strong expression in normal cells but is present in very low concentrations or absent in the cytoplasm of apoptotic cells [21]. A significant increase in the positive expression of Bcl-2 proteins (darker color) was observed in the GCs of normal control rats (Figure 3(a)), the Menoprogen group (Figure 3(c)) and Progynova group (Figure 3(h)), compared with the negative expression in NAM group (Figure 3(b)). Moreover, Bcl-2 expression in the Progynova group was higher than in the Menoprogen group showing that Progynova reduced GC apoptosis better than Menoprogen (Figure 3(d)). Bax is a proapoptotic molecule, and Bax proteins are more highly expressed in apoptotic cells than in normal cells [21]. In this study, the GCs from normal control rats weakly expressed Bax proteins as shown in Figure 3(e) by a lighter color. The NAM rats showed a strong positive expression of Bax as shown with a darker color (Figure 3(f)). The expression of Bax proteins was weakly observed in both MPG-fed (Figure 3(g)) and Progynova-treated rats (Figure 3(h)), respectively. However, Bax protein expression in the Progynova-treated rats declined more than in the Menoprogen-treated animals, again suggesting that estrogen valerate was slightly better at reducing apoptosis than MPG.

### 3.7. Effects of MPG-Medicated Serums on CdCl-Induced Apoptosis in Cultured GCs

The optical density (OD) measurement of GCs isolated and cultured from the NAM group was significantly lower than that of the GCs isolated from normal control rats or those treated with MPG, respectively (Table 4). The results indicate that serum from MPG-fed rats (at the three different doses) protects isolated rat ovarian GCs from CdCl-induced apoptosis. Based on the most optimally protective effect, the middle dose of MPG was used as the medicated serum sample in further experiments.

### 3.8. Effect of Menoprogen Treated Serum on Cultured GC Ultrastructure

**Figure 4:**
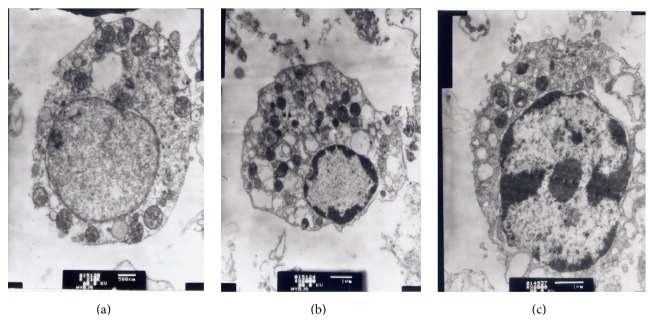
The in vitro effect of Menoprogen-medicated serum on GC ultrastructure. (a) The GCs showed a normal ultrastructure in normal control rats. (b) In the MPG-medicated serum group, both cellular organelles and secretary granule were enriched. (c) In the NAM group, it was observed that chromatin within nucleus was in shifting and there was marginal aggregation, endocytoplasmic reticulum became vacuolated, and the number of secretary granules was decreased.

As assessed by TEM, the GCs had a normal ultrastructure in normal control group (Figure 4(a)). However, the membranes from GCs obtained from untreated NAM rats showed declining or fading microvilli and visible foaming phenomena. In addition, the chromatin within the nucleus showed marginal aggregation with partial fragments or pyknosis. The endocytoplasmic reticulum was vacuolated and the number of secretary granules was reduced (Figure 4(c)). When the GCs were cultured with the MPG-medicated serum, the nucleus exhibited primarily euchromatin, suggesting an increase in active gene transcription. The cellular organelles and secretory granules were enriched, and the tumidity of endocytoplasmic reticulum was also reduced (Figure 4(b)).

### 3.9. FCM Analysis of GC Apoptosis after Treatment with MPG-Medicated Serum

**Figure 5:**
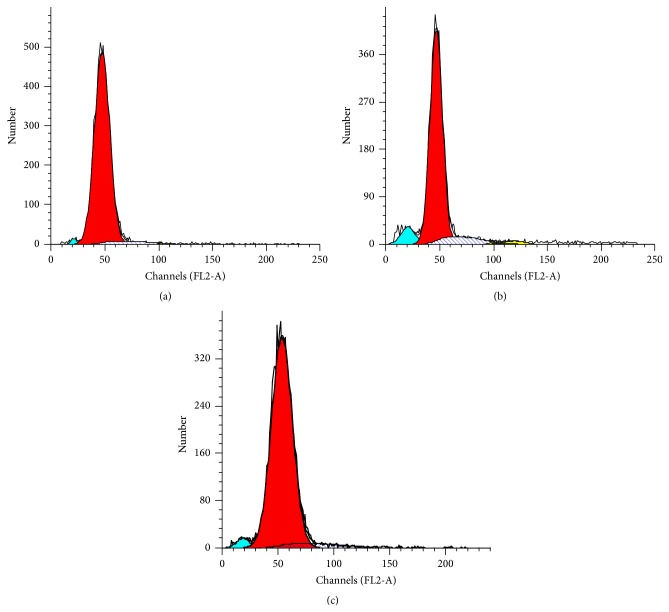
Effect of Menoprogen-medicated serum on peak of CdCl-induced GC apoptosis in vitro. (a) FCM analysis displayed a small apoptotic peak in the cultured GCs isolated from the normal control rats, (b) an obvious apoptotic peak in the GCs isolated from the NAM model, and (c) a >50% reduction in peak size after addition of MPG-medicated serum.

In Figures 5(a)–5(c), the large red peak represents the G_1_ peak, and the blue-green peak is the sub-G_1_ peak. In the DNA histogram, the sub-G_1_ presenting before the G_1_ peak is considered the apoptotic peak. In GCs isolated from normal control rats, FCM analysis shows a very small apoptotic peak and only one main G_1_ peak in the GCs of normal control rats (Figure 5(a)). However, GCs isolated from NAM rats show a large apoptotic peak (Figure 5(b)), which decreased significantly (>50% reduction) after the addition of the MPG-medicated serum to the cultured GCs (Figure 5(c)).

### 3.10. Effects of MPG-Medicated Serum on Rat GC Caspase-3 Protein Expression

Caspase-3 protein (C3P) is a member of the cysteine-aspartic acid protease (caspase) family and plays a central role in cellular apoptosis. The expression of the C3P was significantly higher in the untreated NAM rats, but C3P levels were significantly reduced in cultured GCs after treatment with MPG-medicated serum (Table 5).

## 4. Discussion

As women worldwide look for alternatives to hormone therapy to manage their menopausal symptoms, many questions arise as to the safety and efficacy of these herbal therapies, as well as their potential mechanisms of action [22]. While many plausible mechanisms of action for herbals used for menopause have been proposed, such as the “phytoestrogen” hypothesis, the mechanisms of action for most herbal medicines have remained elusive [22].

The Menoprogen herbal formula tested in this study is an ancient TCM formula that has been used for the management of menopausal symptoms in China for many years [8, 9]. Two preliminary clinical trials have demonstrated that this formula reduced the Kupperman index in menopausal Chinese women and increased endogenous E_2_ levels [8, 9]. However, its mechanism of action has remained unknown. Previous animal studies have shown that MPG increases endogenous E_2_ levels in rats, but further ER-binding studies have shown that MPG does not have direct estrogenic effects, in that it does not act as a phytoestrogen [14, 23, 24]. Since ovarian granulosa cells are the primary source of estradiol in mammals, we hypothesized that the mechanism of this herbal formula may directly involve the ovary and granulosa cells apoptosis.

Granulosa cells play a crucial role in female reproduction by synthesizing sex hormones, including E_2_, as well as interacting with the oocytes [4, 5]. Granulosa cells surround the oocyte and play an important role in its development. Reducing GC apoptosis favors follicular survival [25, 26]. However, as women age, there is a significant reduction in both follicular number and function through follicular atresia, leading to reduction in E_2_ levels and culminating in the menopause [1]. It is this significant reduction of E_2_ levels that precipitates vasomotor and other menopausal symptoms. Since apoptosis is the driving force behind follicle atresia during ageing and causes an age related decline in the function of oocytes and granulosa cells [4–6], we hypothesized that MPG may have antiapoptotic effects on ovarian GCs and thereby improve the function and E_2_ producing capacity of these cells.

Our results demonstrated that when compared with the normal morphology observed in the GCs isolated from young female rats, the nuclei of GCs from NAM rats exhibited smaller cell bodies and more concentrated heterochromatins and apoptotic cells. These results were consistent with other studies in mammalian species showing GC apoptosis as a result of ageing [6, 25]. There was a significant reduction in GC apoptosis in NAM rats treated with varying doses of MPG or estradiol valerate, and more abundant and mature smooth endocytoplasmic reticulum (SERs) were apparent in the GCs of MPG-treated rats. As a comparison, one study involving young women with active estrogen synthesis showed that their GCs typically featured steroidal-secreting cells, that is, less rough endocytoplasmic reticulum (RER) and more SERs, as well as more and larger mitochondria [27]. Furthermore, in the TUNEL assay, untreated NAM rats showed a greater number of atretic follicles, as well as more prominent TUNEL-positive GCs, indicating a significant level of GC apoptosis. However, in the MPG and estradiol valerate treated groups, there were more developing follicles at different stages, indicating a significant reduction in GC apoptosis. The results also showed an abundance of SERs with evident dilation, as well as increased numbers and larger mitochondria in the GC cytoplasm and higher E_2_ levels in rats treated with MPG or Progynova. These data demonstrate that the reduction of GC apoptosis by both treatments involves not only an improvement in GC morphology, but also in their function, as there is an increase in E_2_ synthesis and secretion. Further in vitro data supported the in vivo studies and showed that MPG treatments also reduced CdCl-induced GC apoptosis. CdCl was used as a pathological model based on its ability to induce GC apoptosis and cause harmful effects on the mammalian reproductive system [28]. Thus, in addition to the management of menopausal symptoms, MPG may be useful for the treatment of women who have been exposed to reproductive toxins.

Apoptosis and the regulation of the cell cycle are associated with key control points during different phases of cell cycle (G_1_, S, G_2_, and M) [29]. It has previously been reported that several Traditional Chinese Medicines (TCM) alter apoptosis in various cell lines through the regulation of the cell cycle [30–32]. When we compared untreated NAM rats with those treated with MPG or Progynova, the treated animals exhibited significant decreases in the number of GCs in the G_0_/G_1_ stage of the cell cycle and also showed a progression from G_0_/G_1_ into S stage indicating increased cell division. In the TUNEL assay, the observed increase in apoptotic expression of GCs from NAM rats was almost identical to that observed in other studies [33], further supporting the relationship between follicular atresia and apoptosis. Taken together, the data demonstrate that treatment of the NAM rats with MPG or estradiol valerate reduces GC apoptosis and thereby improves the structure and function of GCs (endogenous E_2_ levels are increased after treatment).

Inasmuch as apoptosis is a highly regulated process, in which cells activate signaling pathways that lead to programed cell death; we also investigated some of the signaling factors well known to be involved in apoptosis, namely, the Bcl-2 and caspase family proteins [6, 34]. The Bcl-2 family proteins are located in the outer mitochondrial membrane, the endocytoplasmic reticulum membrane, and the nuclear membrane and are key regulators of apoptosis whose action site is the mitochondrial membrane [6, 34, 35]. This family includes the antiapoptotic proteins Bcl-2 and Bcl-x, with Bcl-2 being the first cell survival factor discovered in mammals [34, 35]. A reduction of Bcl-2 expression results in cell apoptosis, thereby demonstrating that it plays a critical role in its regulation [34]. Sasson et al. [35] also found that Bcl-2 levels were up to 3–7.6 times higher when human GC apoptosis was inhibited by corticosteroids. They further demonstrated that inhibition of Bcl-2 leads to the inhibition of GC apoptosis, as well as the stimulation of follicular growth and ovulation by Bcl-2 [35]. In another study, Kugu et al. [36] demonstrated an increase in the expression of the proapoptotic protein, Bax, in the GCs of atretic follicles at an early stage, but a lower expression of Bax in normal follicles or in old atretic follicles, linking the stimulation of Bax proteins to the early stages of GC apoptosis in atretic follicles [6, 36]. In addition, the ratio of Bcl-2 to Bax in the dimer impacts the apoptotic status. If the ratio of Bcl-2 > Bax, the dimer prefers cell survival; if the ratio of Bcl-2 < Bax, the apoptosis would be dominant [37, 38]. In this work, we demonstrate that in vitro cultured GCs treated with MPG or estradiol valerate (Progynova) exhibited an increased expression of Bcl-2 proteins and reduced the expression of proapoptotic Bax proteins, thereby shifting the balance to favor GC survival.

Finally, in addition to Bcl-2 family of proteins, the caspases are also known to play a central role in the process of apoptosis and are members of the cysteine-aspartic acid protease (caspase) family [39]. During apoptosis, after the release of cytochrome C from the mitochondria, apoptosis activating factor and caspase-9 mediate the signal that activates caspase-3 [6]. The activation of caspase-3 leads to the alteration in the expression of the Bcl-2 family proteins [6]. The sequential activation of caspases is critical for the execution phase of cell apoptosis in GCs [39]. Several previous reproductive endocrinology studies have demonstrated that there is an involvement of caspase proteins in GC apoptosis [39, 40]. For example, activated caspase-3 is present in oocytes and GCs of rats and plays a role in GC apoptosis and follicular atresia [39, 40]. Lzawa et al. [41] confirmed the presence of genomic transcripts and precursor proteins of caspase-3 in GCs by PCR and Western blotting. Our results found a significant increase of caspase-3 levels in the GCs isolated from NAM rats as compared with normal young rats, further supporting the relationship between caspase-3 and GC apoptosis. Based on our results showing a decrease in the caspase-3 concentration in GCs from Menoprogen-treated animals, it is suggested that Menoprogen may reduce GC apoptosis by blocking the caspase cascade and altering the expression of Bcl-2 and Bax proteins.

In terms of going forward with future clinical studies, the results presented here indicate that the low or middle doses of 162–324 mg may have a more optimal effect than the high dose, which is not uncommon for herbal medicines. The low dose had a more significant effect on the uterine and ovarian coefficients, as well as increasing the number of GCs in the S phase of the cell cycle, while the middle dose had the best effect on E_2_ levels. Since the increase of endogenous E_2_ would most likely lead to menopausal symptom reduction, the use of the middle dose would likely be logical. Further support for the use of the middle dose comes from a previous clinical study that treated menopausal women with 200 mg of MPG and demonstrated a significant reduction in the KMI and menopausal symptoms, as well as increases in serum E_2_ and P4 [8]. However, this study was not a randomized controlled clinical trial (RCT), and a larger RCT should be performed to determine safety and efficacy.

In conclusion, Menoprogen, a TCM formula for treating menopausal symptoms appears to have a novel mechanism of action in that it is not directly estrogenic but instead works indirectly by reducing GC apoptosis, which in turn improves the function of GCs, thereby enhancing their capacity to synthesize and secrete E_2_. Unlike estradiol valerate, Menoprogen had no direct estrogenic effects and does not stimulate the proliferation of breast cancer cells in vitro [23]. Thus, this represents a very novel mechanism of action for this herbal formula.

## Figures and Tables

**Table 1:** Effects of Menoprogen and estradiol valerate (Progynova) on ovarian and uterine weight of aged rats ($\overset{-}{x}\pm sd$).

Rat group	Ovarian coefficient (10^−1^)^a^	Uterine coefficient (10^−1^)^a^
Normal control	2.79 ± 0.38^*∗∗*^	2.06 ± 0.55^*∗∗*^
NAM	2.15 ± 0.46	1.47 ± 0.23
Low-dose	2.70 ± 0.34^*∗∗*^	1.72 ± 0.25^*∗*^
Middle-dose	2.56 ± 0.37^*∗*^	1.99 ± 0.23^*∗∗∗*^
High-dose	2.63 ± 0.37^*∗*^	1.97 ± 0.48^*∗∗*^
Progynova	2.51 ± 0.58	1.94 ± 0.53^*∗*^

^*∗*^ *P* < 0.05, ^*∗∗*^ *P* < 0.01, and ^*∗∗∗*^ *P* < 0.001 versus NAM group.

^a^Organ coefficient = organ wet weight/body weight × 100%.

**Table 2:** Effects of treatments on serum E_2_ levels in NAM rats as compared with normal 4-month-old female rats and untreated NAM rats ($\bar{x}\pm sd$) (*n* = 10 per arm).

Rat group	E_2_ (pg/mL)	
	Before treatment	After treatment
Normal control	72.80 ± 28.29	69.78 ± 19.68^*∗∗∗*^
NAM (untreated)	38.23 ± 11.50^*∗∗*^	39.68 ± 13.32
Low-dose	45.17 ± 15.63^*∗∗*^	66.41 ± 14.27^*∗∗∗*^
Middle-dose	48.81 ± 27.63^*∗∗*^	92.50 ± 23.88^*∗∗∗*^
High-dose	40.84 ± 10.17^*∗∗*^	70.50 ± 9.34^*∗∗∗*^
Progynova	42.01 ± 13.07^*∗∗*^	71.36 ± 8.96^*∗∗∗*^

^*∗∗*^ *P* < 0.01 versus normal control group; ^*∗∗∗*^ *P* < 0.001 versus NAM group.

**Table 3:** Effects of Menoprogen on GC cell cycle ($\overset{-}{x}\pm sd$) (*n* = 10 rats per arm) as determined by FCM.

Rats group	G_0_/G_1_	S	G_2_/M
Normal control	82.60 ± 4.56^*∗*^	9.80 ± 2.83^*∗*^	8.40 ± 2.00^*∗*^
NAM	91.18 ± 1.44	4.70 ± 2.07	4.13 ± 1.19
Low-dose	87.72 ± 1.29	11.08 ± 4.68^*∗*^	3.78 ± 1.10
Middle-dose	83.65 ± 1.57^*∗*^	8.07 ± 1.96^*∗*^	4.86 ± 1.02
High-dose	87.07 ± 0.97^*∗*^	5.96 ± 2.36	5.27 ± 2.75
Progynova	88.83 ± 3.66	7.67 ± 1.24	4.61 ± 0.75

^*∗*^ *P* < 0.05 versus NAM group.

**Table 4:** The effect of serum from Menoprogen-fed rats on in vitro GC apoptosis ($\overset{-}{x}\pm sd$) (*n* = 8 per arm) using the MTT method. The final results were performed in triplicate and repeated three times.

Rat group	Optical density
Normal control	0.54 ± 0.04^*∗∗*^
NAM	0.45 ± 0.04
Low-dose medicated serum	0.62 ± 0.07^*∗∗*^
Middle-dose medicated serum	0.72 ± 0.07^*∗∗*^
High-dose medicated serum	0.51 ± 0.04^*∗∗*^

^*∗∗*^ *P* < 0.01 versus NAM group.

**Table 5:** Effect of MPG-medicated serum on caspase-3 protein expression in rat GCs (*μ*mol/L) ($\overset{-}{x}\pm sd$) (*n* = 3).

Rat group	Caspase-3
Normal control	72.55 ± 1.70^*∗*^
NAM rats	82.14 ± 3.45
Menoprogen-fed serum	56.46 ± 7.49^*∗*^

^*∗*^ *P* < 0.05 versus model group.
